# Chicago Pathological Society—Secretary’s Report

**Published:** 1883-07

**Authors:** J. H. Tebbetts

**Affiliations:** Secretary


J. H. TEBBETTS, Secretary.

---

### ARTICLE XIII.

### CHICAGO PATHOLOGICAL SOCIETY.—SECRETARY'S REPORT.

MEETINGS.—The first meeting of the year, the annual meeting, was held May 1, 1882, and the last April 9, 1883. The whole number of meetings during the year was ten. Two meetings were without a quorum, owing to meetings of other societies on the same evening.

MEMBERSHIP.—One hundred and twenty-four members have attended during the year; an average of about twelve at each meeting.

NEW MEMBERS.—Sixteen gentlemen and ladies were proposed for membership during the year, all of whom were unanimously elected, making the total membership at the end of the year one hundred and two.

POSTALS.—There have been printed and mailed to members 946 postal cards, giving time and place of meeting and order of exercises.

WORK OF THE YEAR.—The following literary and scientific work has been accomplished by the society during the year:

Nine papers; six detailed reports of particularly interesting cases; two exhibitions of microscopical preparations; one announced discussion;—and were entitled as follows:

1. Paper.—Dr. John A. Robinson. "Medical Geography of California."

2. Verbal Report.—Dr. G. S. Campbell. "Northern Rocky Mountain Country."

3. Paper.—Dr. E. P. Davis. "Nerve Stretching."

4. Paper.—Dr. G. Frank Lydston. "Treatment of Erysipelas."

5. Paper.—Dr. H. D. Valin. "Sunstroke."

6. Case Report.—Dr. Joseph Haran. "Hypertrophy of Heart," with specimen.

7. Case Report.—Dr. Odelia Blinn. "Post-partum Hæmorrhage."

8. Paper.—Dr. C. W. Earle. "Cirrhosis of Pancreas."

9. Paper.—Dr. Adelia Barlow. "Tetanus."

10. Case Report.—Dr. H. M. Lyman. "Locomotor Ataxia, treated by dry cupping."

11. Exhibition of Microscopical Specimens.—Dr. Belfield. "Bacillus of Tuberculosis."

12. Case Report.—Dr. J. H. Tebbetts. "Cancer of Stomach." Scirrhus, with specimens.

13. Paper.—Dr. H. M. Lyman. "Sleep and its Disorders; Hypnotism, Somnambulism, etc."

14. Case Reports.—Dr. G. F. Lydston. Two cases.

15. Exhibition, Microscopical Specimens.—Dr. W. T. Belfield. "Cultivation Anthrax Bacilli in Gelatine."

16. Paper.—Dr. G. Frank Lydston. "A Contribution to the Hereditary and Pathological Aspects of Crime."

16. Paper.—Dr. A. Lagorio. "The Hypodermic Treatment of Syphilis."

18. Paper.—Dr. G. F. Lydston. "The Treatment of Bubo."

19. Discussion.—"Rheumatism," opened by Dr. C. J. Lewis.

At the September meeting, the society learned of the recent sad bereavement of the secretary, Dr. R. S. Hall, whose resignation was received and accepted at that time. Resolutions of sympathy were passed by the society. At this meeting, the present secretary was elected to serve the remainder of the year.

RESIGNATIONS.—But one member has resigned from membership—Dr. E. Ingals.

SUGGESTIONS.—It would perhaps be a good idea to have our list of members revised and printed in pamphlet form, together with our Constitution. The expense would be slight, and the benefit considerable.

In conclusion, we cannot but note the continued progress and prosperous condition of our Pathological Society; and as our membership increases, our work will of necessity limit itself, perhaps, more closely to pathological work, but at present, we have reason for satisfaction with our year's work and progress.

Respectfully submitted,

